# Influence of Different Rates of Plant-Based Compost on Clay Soil Metal Behavior and Human Health Risk Assessment in *Moringa oleifera* Leaf Biomass

**DOI:** 10.1007/s00128-024-03894-x

**Published:** 2024-05-09

**Authors:** N Ngwenya, Y Nuapia, I Risenga, L Chimuka

**Affiliations:** 1 School of Animal, Plant, & Environmental Science, University of Witwatersrand, Johannesburg, South Africa; 2 Pharmacy Department, School of Healthcare Sciences, University of Limpopo, Polokwane, South Africa; 3 Molecular Sciences Institute, School of Chemistry, University of Witwatersrand, Johannesburg, South Africa

**Keywords:** Organic soil amendment, Nutrient bioavailability, Accumulation

## Abstract

An investigation of the impact of adding plant-based organic compost to clay soil from a *Moringa oleifera* farm focusing on the metal content, bioavailability, and accumulation of nutrients in *M. oleifera* leaves was conducted. Clay soil was mixed with 15%, 30%, 45% and 60% plant-based organic compost (by volume) in 20 cm wide, 2 L pots. *Moringa oleifera* plants were planted in four replicates of each treatment and control group. Results revealed that the addition of compost significantly (*P* < 0.05) altered the concentration of metals in the soil. Correspondingly, accumulation of nutrients in *M. oleifera* leaves increased with the addition of compost to the soil, except for cobalt and chromium. Trace elements had minimal bioavailability in the amended soils, and their presence in the leaves was lower than the permissible trace metal levels in food. The 30% combination had the highest concentration of calcium (45 042.5 mg/kg), magnesium (17430.0 mg/kg) and phosphorous (8802. 5 mg/kg) in *M. oleifera* leaves. The study concluded the addition of compost improved bioavailability of nutrients in the soil and their concentration in *M. oleifera* leaves. The target hazard quotients for heavy metals was less than one, indicating that *M. oleifera* leaf biomass harvested from soil amended with plant-based compost is safe for human consumption. These results serve as guidelines for recommended organic certification requiremets where plant-based compost is often used in the fast-growing herbal industry.

## Introduction

*Moringa oleifera* L. commonly known as “horseradish,” “drumstick,” or “miracle tree” is a soft-stem plant in the monogeneric Moringaceae family (Gopalakrishnan et al. 2016). Native to the Himalayan Mountains of India, Pakistan and Nepal (Leone et al. 2015), it also grows naturally in Namibia, Angola, Kenya, Ethiopia and Egypt (Konmy et al. 2016). *Moringa oleifera* is well-known for its anti-inflammatory, anticancer, anthelmintic and antiurolithiatic properties, and it also helps to alleviate and prevent cardiovascular ailments (Bhattacharya et al. 2018). Leaves are the most commonly used part of the plant, containing more vitamin C (400 mg) than oranges (30 mg) per 100 g biomass (Mahmood et al. 2010). Leaves also contain vitamins B, D and E; nicotinic acid; pyridoxine (Siddhuraju and Becker 2003); copper (Cu), zinc (Zn), iron (Fe), magnesium (Mg) and potassium (K) (Aslam 2005).

Global awareness of *M. oleifera* as a nutraceutical has increased, leading to its increased utilization and production (Brilhante et al. 2017). Production is expected to increase steadily as demand for a healthier lifestyle, organic foods, and nutritional supplements grows (Akinola et al. 2020). In South Africa, *M. oleifera* production for local and international markets is increasing steadily, with farms present in Limpopo, Northwest, Gauteng, KwaZulu-Natal, Western Cape and Mpumalanga Provinces (Mashamaite et al. 2021). Cultivation, climate, location, and soil physiochemical properties affect production and nutrient content of *M. oleifera* (Grosshagauer et al. 2021). Artificial fertilizers have been used to increase the production and nutrient content of *M. oleifera* (Sarwar et al. 2017). Despite increasing growth, artificial fertilizers may result in the accumulation of heavy metals in plant leaves (Chibuike and Obiora 2014). Furthermore, as consumers gradually embrace a heathier lifestyle, demand for organically grown food has steadily increased (Meemken and Qaim 2018). Thus, many *M. oleifera* farmers are avoiding the use of artificial fertilizers.

Compost is an organic fertilizer that supports sustainable agriculture. It improves the physiochemical, organic matter, and nutrient content of the soil (Adugna 2016). Organically grown plant products may contain higher levels of antioxidants, minerals, and dry matter, and have less chemical residues (Crinnion 2010). Studies perfomed in Nigeria revealed that organically grown *M. oleifera* plants performed better in terms of biomass and the nutritious status compared to the control and nitrogen (N), phosphorous (P), and K (NPK) fertiliser treatments (Adebayo et al. 2017). Use of compost in the production of *M. oleifera* improved the nutritional content of the plant and also helped prevent soil degradation (Ngakou et al. 2020). Studies conducted in South Korea revealed that a combination of general compost and NPK improved the biochemical content of *M. oleifera* (Sarwar et al. 2020). Organic soil amendments widely used by farmers are generally from livestock manure. However, previous research has revealed that improperly treated and utilized livestock manure may pose a health risk to the environment due to microbial contamination and addition of some chemicals to animal feed (Kumar et al. 2013). Foodborne pathogenic bacteria such as *Salmonella* and *Escherichia coli* were detected in agricultural soils and in vegetables grown with animal manure application (You et al. 2006). In addition, compost obtained from sewage sludge and livestock excreta can contain traces of heavy metals (Liu et al. 2006).

Compost obtained from plant-based material has been used as a fertilizer in organic farming, as it contains little to no heavy metals (Bożym 2017) and fecal coliforms. Plant-based compost also suppressed plant diseases caused by the pathogenic formae speciales *Fusarium oxysporum* (Yogev et al. 2006). Compost is used for soil amendment programs to reduce heavy metal contamination (Huang et al. 2016). Soil amendments are essential in *M. oleifera* farms around South Africa (Mashamaite et al. 2021), as approximately 50% of the land is deemed less than suitable for *M. oleifera* production based on climate and soil physiochemical properties (Tshabalala et al. 2020). This study focused on organic soil amendments to improve the nutrient content of *M. oleifera* from the community farm in Hammanskraal, Gauteng, South Africa. Soils in this area pose a challenge to *M. oleifera* growth as they are prone to water logging. The soil also cracks when dry, exposing *M. oleifera* tubers to frost during winter. Plant-based compost amendments are expected to improve soil physiochemical properties and nutrient quality of *M. oleifera*. Limited scientific literature on the use of plant-based compost to increase the nutrient quality of *M. oleifera* leaves grown in clay soil exists. In addition, no human health risk assessment has been carried out on the potential hazard associated with consuming compost grown *M. oleifera* products.

## Materials and Methods

### Plant Material, Treatments and Sowing

*Moringa oleifera* seeds and the clay soil used were obtained from the community farm in Hammanskraal, Gauteng, South Africa. Topsoil (0–15 cm) was collected using a spade, air-dried, homogenized, and sieved (0.25 μm mesh) to remove gravel and debris. Seeds were primed by soaking in water for 24 h and subsequently planted into germination trays containing potting soil and watered every two days for a period of four weeks. Four weeks old seedlings exhibiting uniform optimum growth were then transferred into 20 cm diameter, 2 L plastic pots containing clay soil as a control and clay soil amended with four levels (85% soil: 15% compost; 70% soil: 30% compost; 55% soil: 45% compost and 40% soil: 60% compost) of organic plant-based compost as treatments in four replicates each. The percentage of each compost-soil combination was calculated by the volume of the total mixture. Plants were watered with tap water twice a week, after which plant growth was monitored for six months. Treatment percentages followed those used by Vouillamoz and Milke (2001) and Zheljazkov and Warman (2004). Plants were grown in the greenhouse at the School of Animal, Plant and Environmental Sciences (APES), University of the Witwatersrand under ambient temperature.

### Sample Preparation

All leaves in each replicate were harvested and washed, and both leaf and soil samples were air-dried under the shade, ground and sieved. Processed samples were stored in polyethylene bottles. Soil pH and electrical conductivity (EC) were determined by a pH and conductivity meter after suspending soil in deionized water at a ratio of 1: 2 soil (20 ± 0.001 g) and deionized water (40 mL), respectively.

### Metal Content in Soil and in *M. oleifera* Leaves

#### Microwave Sample Digestion and Metal Content Analysis

A microwave digester (Anton Paar, Switzerland) was used for digestion. A mass of 0.1 ± 0.001 g sieved soil (0.25 μm mesh) was weighed into a Teflon microwave digestion vessel, after which 9 mL of concentrated HCl, 3 mL concentrated HNO_3,_ 1 mL concentrated HF and 6 drops of concentrated H_2_O_2_ were added. Digestion was then performed at 200 °C for 30 min. To a mass of 0.1 ± 0.001 g dried leaf powder in a Teflon microwave digestion vessel was added 8 mL of concentrated HNO_3_ and 2 mL of concentrated H_2_O_2_. Microwave digestion was carried out for 30 min. Each sample was digested in triplicate.

The digested solution for both soil and leaf samples was transferred into a 50 mL volumetric flask, and the solution was mixed with deionized water. Digested samples were then filtered, and dilutions were made before analyses of the metal content using inductively coupled plasma – optical emission spectroscopy (ICP-OES) (Spectro Genesis, Spectro, Germany). A carbon (C), hydrogen (H), N and sulphur (S) analyzer (CHNS) was used to determine the percentages of each element.

#### BCR Sequential Extraction of Metals

Bioavailability of metals in the soil was determined by slightly changing the modified BCR sequential extraction method of Alan and Kara (2019). The procedure was applied to 1 ± 0.001 g of each soil sample placed in 50 mL centrifuge tubes, and each soil sample was replicated thrice. The procedure is summarized as follows.

In the first step (exchangeable fraction), 40 mL of 0.11 molL^− 1^ acetic acid was used to extract free and acid-soluble fractions bound to carbonates. The sample was shaken at 30 ± 10 rpm for 16 h at room temperature, 25 ± 1 °C. The extract was separated from the solid by centrifuging at 3000 rpm for 20 min, collected and stored in polyethylene bottles at 4 °C. Washing was performed with 20 mL of deionized water, and the mixture was shaken for 15 min and subsequently centrifuged at 3000 rpm. The supernatant was discarded. The second step focused on the reducible fraction, i.e., extraction of metals bound to Fe and Mn oxides. To the residue from step one, 40 mL of freshly prepared 0.5 molL^− 1^ hydroxy ammonium chloride (NH_2_OH. HCL) at pH 1.5 (adjusted by adding 25 ml of 2 molL^− 1^ HNO_3_) was added. The mixture was shaken for 16 h at room temperature. Separation of the extract and washing were performed as described in step one. In the third step (oxidizable fraction, bound to sulfides and organic matter), 10 mL of 8.8 molL^− 1^ of concentrated H_2_O_2_ of pH 2–3 (pH adjusted by adding HNO_3_) was added to the step 2 residue. The mixture was digested for an hour at room temperature. Digestion was continued for another hour by heating the vessel at 85 °C in a water bath until the residue was reduced to 2–3 mL. Afterwards, 50 mL of 1 moL^− 1^ ammonium acetate (C_2_H_7_NO_2;_ pH 2 adjusted by HNO_3_) was added, and the mixture was shaken for 16 h at room temperature. Extraction and washing of the residue was carried out as described in step 1. Residue was subjected to aqua regia digestion. Total metal content was analyzed using the (ICP-OES) (Spectro Genesis, Spectro, Germany).

### Data Analysis

All measurements were performed in triplicate, and the data was subsequently analyzed using one-way analysis of variance (ANOVA) with the SAS statistical package version 17.0. Significance within the means was compared using Fisher’s test.

### Human Health Risk Assessment

The Target Hazard Quotient (THQ) estimates the possible carcinogenic risk associated with the consumption of *M. oleifera* leaf biomass and was determined by the equation:

$${\text{THQ}}= \frac{EF\times ED\times DIM}{RfD\times W\times T}$$

where EF is the exposure frequency (365 days/year); ED is the exposure duration in a lifetime; DIM is the daily metal intake; RfD is the reference oral dose of metals in mg/kg/day with values of 1, 0.02, 0.001, 0.7, 0.00005 and 0.003 for Al, Co, Cr, Fe, Mn and Zn, respectively; W is the average body weight for the Johannesburg South Africa population, set at 62.4 kg and 70.84 kg for males and females respectively, (Nuapia et al. 2018) and T is the average time for noncarcinogenic exposure (365 days/year × number of exposure years). The exposure duration was estimated to be 59.1 and 63.1 for male and female residents respectively, in Johannesburg, South Africa (Nuapia et al. 2018). The DIM was calculated by multiplying the concentration of metals with the recommended daily dose (40 g) of *M. oleifera* leaf powder (Asiedu-Gyekye et al. 2014).

## Results and Discussion

### Soil pH, Electrical Conductivity, and Carbon and Nitrogen Content

The pH of the control clay soil was 7.68 while that of the compost was 8.01 (Table S1). However, the addition of compost to the soil slightly reduced the pH (Table S1). Similarly, the EC of the compost (249.67 µS/cm) was greater than that of the control clay soil (14. 62 µS/cm) (Table S1). The total C and N contents in the soil increased with the addition of compost, as expected (Hu et al. 2018). Although the C and N contents increased simultaneously, the C: N decreased with the addition of compost to the soil. A C: N lower than 20 for all the soil amendments indicated very stable organic matter (Ammari et al. 2012) and the ability of the plants to assimilate N. These values are ideal for nutrient uptake, as N can be assimilated only when the C: N value is less than 20 (Khwairakpam and Bhargava 2009). High soil electrical conductivity (EC) has been known to increase absorption and accumulation of metals in plant shoots (Salimi et al. 2012; Pakade et al. 2013). Addition of compost to clay soil increased the conductivity because of increased cation exchange capacity (CEC) (Sung et al. 2011). Duong et al. (2012) also discovered that addition of compost to clay soil increased EC and N availability to wheat plants in comparison with unamended clay soil.

### Total Metal Concentration in Clay Soil Amended with Plant-Based Compost

Metal concentrations in clay soil amended with compost are presented in Table 1. Plant-based compost had a higher mean concentration of Zn, K, P, S, Na and Ca than clay soil (Table 1). This is expected because compost is made up of plant material, and these are major nutrients found in plants (Hocking 1994). On the other hand, the concentrations of metals needed in trace amounts by plants, such as Al and Fe, were slightly more in clay soil, and the addition of plant-based compost reduced the presence of these elements. High levels of metals (e.g. Fe concentration of 28629.2 mg/kg) were also detected in compost by Manungufala et al. (2008). The high increase in P in the soil upon the addition of compost was due to the high P levels in the plant compost, consistent with the findings of Verma et al. (2013) where compost increased the solubility and concentration of P in the soil. Thus, the addition of compost to clay soil generally increases the concentration of metals present in high amounts in compost.

**Table 1:** Metal concentrations (mg kg^− 1^, mean ± SD, *n* = 3) in clay soils amended with different levels of plant-based organic compost

	Compost	Control (clay soil)		Clay soil + 15% compost	Clay soil + 30% compost	Clay soil + 45% compost	Clay soil + 60% compost
Al	13962.5 ± 56.8^a^		65700.0 ± 1397.0^b^	62075.0 ± 1438.6^c^	52520.0 ± 1223.2^d^	63687.5 ± 1683.7^bc^	58225.0 ± 1213.8^e^
Ca	36607.5 ± 907.5^a^		13162.5 ± 555.0^b^	13730.0 ± 109.3^b^	11980.0 ± 381.3^c^	15355.0 ± 442.5^d^	15432.5 ± 596.8^d^
Co	59.3 ± 0.8^a^		83.5 ± 1.0^b^	81.7 ± 0.6^c^	79.3 ± 1.2^d^	82.8 ± 0.3^bc^	79.5 ± 0.5^d^
Cr	106.3 ± 1.8^a^		227.5 ± 2.6^b^	226.5 ± 2.3^b^	216.2 ± 5.4^c^	227.3 ± 0.8^b^	200.5 ± 2.2^d^
Fe	12425.0 ± 65.5^a^		46487.5 ± 638.2^b^	43937.5 ± 809.7^c^	42497.5 ± 967.3^d^	46265.0 ± 990.0^b^	39070.0 ± 657.5^e^
K	12460.0 ± 321.9^a^		5755.0 ± 114.6^b^	6215.0 ± 351.2^c^	5835.0 ± 217.0^b^	7015.0 ± 341.3^d^	6700.0 ± 371.8^cd^
Mg	5444.8 ± 26.1^a^		6209.7 ± 61.8^b^	6210.8 ± 58.1^b^	6018.0 ± 161.6^c^	6626.8 ± 9.0	6273.0 ± 76.0^b^
Mn	613.5 ± 4.5^a^		672.0 ± 1.3^b^	687.0 ± 1.8^bc^	665.3 ± 16.8^d^	757.7 ± 3.7^e^	699.5 ± 11.4^c^
Na	1904.8 ± 26.1^a^		479.0 ± 69.7^b^	500.7 ± 3.3^b^	599.2 ± 51.0^c^	745.8 ± 14.9^d^	591.0 ± 34.4^c^
P	7052.5 ± 45.2^a^		2765.0 ± 4.3^b^	2565.0 ± 45.6^c^	2805.0 ± 79.0^b^	2890.0 ± 30.3^d^	3422.5 ± 38.5^e^
S	5457.5 ± 126.0^a^		2630.0 ± 104.0^b^	3040.0 ± 99.9^c^	3557.5 ± 232.5^d^	3832.5 ± 41.8^e^	3615.0 ± 136.5^de^
Zn	277.8 ± 1^a^		209.3 ± 7.5^b^	243.8 ± 0.8^c^	234.7 ± 6.1^d^	256.3 ± 2.9^e^	266.5 ± 5.1^f^

Different letters in each row indicate significant difference (*P* < 0.05).

### Total Concentrations of Metals in Dried *M. oleifera* Leaves

Metal concentrations of dried *M. oleifera* leaves are shown in Table 2. Concentrations of macro- and micronutrients in *M. oleifera* leaf powder varied significantly (*P* < 0.05) with the addition of compost to the soil, with the exception of Co and Cr (Table 2). Variation was also noted within different levels of plant-based compost (Table 2). Similarly, compared with plants of other compositions, leaf biomass of plants grown in clay soil amended with 30% compost had the highest concentrations of metals, approximately 50%. Interestingly, the high concentration in 30% compost was attributed to major nutrients such as Ca, Mg, P and S. Adebayo et al. (2017) reported an increase in *M. oleifera* leaf metal content upon the addition of different forms of compost, which was similar with our current findings. Clay soil amended with 45% compost contained more Zn. Sodium was highest in the 30% and 45% clay soil compost amendments and leaves from clay soil amended with 60% compost contained more Fe. However, the concentration of Al in the leaves was greatest in the control. This could be due to the fact that addition of compost to soil reduces exchangeable Al and its concentration in plants (Ho et al. 2022). These variations in uptake of metals by *M. oleifera* plants grown in different soil amendments reflect the ability of plant-based compost to alter soil physiochemical properties, which in turn alters metal uptake by roots (Celik et al. 2004). The addition of compost to clay soil improves aeration, water holding capacity and cation exchange capacity (CEC) and thus improves the uptake of metals (Omara et al. 2022). Haouvang et al. (2017) reported that high levels of compost in the soil may lead to toxicity due to overaccumulation of metals such as Fe and Mn.

**Table 2:** Metal concentration (mg kg^− 1^, mean ± SD, *n* = 3) of *Moringa oleifera* leaves harvested from clay soil amended with different levels of plant-based organic compost Different letters in each row indicate significant difference (*P* < 0.05)

	Control (clay soil)	Clay soil + 15% compost	Clay soil + 30% compost	Clay soil + 45% compost	Clay soil + 60% compost
Al	163.0 ± 5.5^a^	91.0 ± 6.5^bd^	113.0 ± 3.6^c^	81.8 ± 4.1^d^	96.0 ± 6.4^b^
Ca	30405.0 ± 1102.6^a^	33400.0 ± 498.4^b^	43042.5 ± 1000.5^c^	22732.5 ± 608.1^d^	30387.5 ± 1396.1^a^
Co	52.0 ± 1.3^a^	52.8 ± 1.0^a^	51.3 ± 1.6^a^	52.3 ± 0.3^a^	51.3 ± 1.6^a^
Cr	101.5 ± 1.3^a^	96.0 ± 1.7^b^	101.0 ± 0.0^ac^	98.0 ± 1.3^bc^	102.0 ± 0.5^a^
Fe	208.5 ± 2.2^a^	223.2 ± 1.8^b^	204.0 ± 2.2^c^	181.0 ± 0.9^d^	265.0 ± 0.5.0^e^
K	13477.5 ± 679.3^a^	18392.5 ± 244.9^b^	28017.5 ± 581.3^c^	16685.0 ± 223.8^d^	30772.5 ± 765.7^e^
Mg	11837.5 ± 414.0^a^	12447.5 ± 72.8^b^	17430.0 ± 212.7^c^	7482.5 ± 256.6^d^	11457.5 ± 303.9^e^
Mn	101.7 ± 0.8^a^	129.8 ± 0.6^b^	140.2 ± 1.1^c^	116.0 ± 0.0^d^	110.2 ± 0.3^e^
Na	1155.8 ± 26.0^a^	1190.7 ± 17.0^a^	1261.5 ± 24.8^b^	1470.5 ± 7.4^c^	1469.5 ± 17.3^c^
P	5330.0 ± 20.3^a^	7215.0 ± 83.5^b^	8802.5 ± 75.9^c^	4062.5 ± 37.0^d^	5507.5 ± 15.6^e^
S	13965.0 ± 81.1^a^	23225.0 ± 22.9^b^	37460.0 ± 69.1^c^	16302.5 ± 97.9^d^	23285.0 ± 172.1^b^
Zn	57.5 ± 0.9^a^	80.2 ± 3.1^a^	62.8 ± 2.0^c^	50.5 ± 0.5^d^	65.2 ± 0.3^c^

### Studying Metal Bioavailability in Clay Soil Amended with Compost

Results for metal speciation in the clay soil and in the plant-based compost treatments are summarized in Figure S1. Increases in the exchangeable forms of Ca, K, Mg, Mn, Na, P and Zn increased with the addition of compost to the soil; however, there was no significant difference (*P* > 0.05) in the different soil to compost ratios. Addition of compost increased the availability of Al and Fe by decreasing the residual fraction and increasing the oxidizable fraction. No significant difference (*P* > 0.05) was detected among the clay soils, and no significant differences were detected for Cr or Co. The calcium exchangeable fraction was greater in the 30% compost clay‒soil amendment, while the Mg and Mn concentrations were greater in both the 15% and 30% treatments. The potassium exchangeable fraction was greatest in the 15% compost clay‒soil treatment group (Figure S1).

Sungur et al. (2014) used sequential extraction to reveal that some metals are present in the soil but are not accessible to plants. Similarly, in the present study, the Al, Fe and Zn concentrations were high in the soil but low in *M. oleifera* leaves (Tables 1 and 2). This could be due to the high soil pH, which increases the ability of Al to bind to plants, decreasing the availability of Al (Zhao and Shen 2018). Zinc is also known to be largely bound to silicate in the soil (Ptistišek et al. 2001). Higher concentrations of Cu and Mn in the residual fraction indicate the immobile state of these metals, and the same trend was reported by Sahito et al. (2015). Furthermore, Ca is highly soluble in water (Van Herck et al. 2000) and acid, as indicated by its high concentration in the acid-soluble fraction (Figure S1 (a), facilitating plant uptake and leading to high Ca concentrations in the leaves. Jakubus (2016) suggested different soil compositions have an effect on the release and phytoavailability of metals. Additionally, organic amendments influence the content and mobility of metals in the soil and the uptake of nutrients by plants (Herencia et al. 2008). The current study affirms these findings.

### Assessment of Possible Adverse Health Effects Posed by Daily Intake of *M. oleifera*

The daily intake of the metals found in the present study is of paramount importance for preventing cumulative toxicity (Grosshagauer et al. 2021). The DIM results (Table S2) indicate that the Al, Fe, Mn and Zn DIM are below the recommended daily intake. However, the Co and Cr concentrations (Table S2) were above the recommended daily intake limits. High levels of Cr could be attributed to the fact that South Africa geologically has high Cr deposits (Nuapia et al. 2018). The concentration of Cr in *M. oleifera* leaf powder from farms around South Africa reported by IDC (2019) was also above the permissible limit of Cr concentration in plants. However, further investigations are needed to determine the sources of Co and Cr in the present study, as the addition of compost did not significantly increase their bioavailability in the soil or their accumulation in *M. oleifera* biomass (Figure S1 (a), Table 2).

**Fig. 1:**
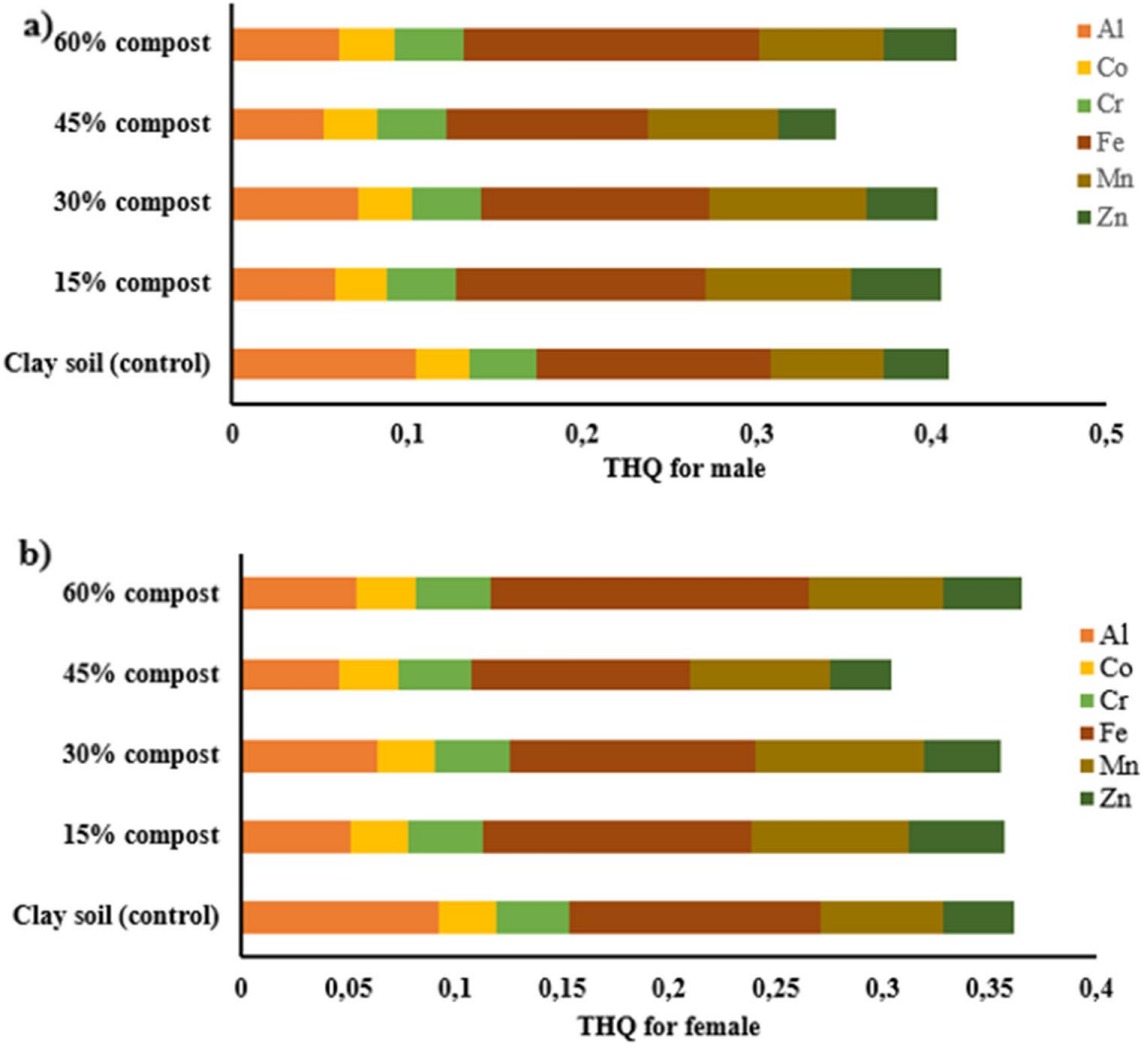
**(a and b)** Target hazard quotients (THQ) for male and female for *Moringa oleifera* leaf biomass harvested from clay soil amended with different levels of plant-based compost

The calculation of health risk assessments for all consumed products is of paramount importance because foodborne toxicity is a global crisis (Areo and Njobeh 2021). In addition, heavy metal contamination has always been a major factor compromising the quality and safety of herbal plants (Okem et al. 2014). Figure 1 shows the noncarcinogenic THQs for Al, Co, Cr, Fe, Mn and Zn.

The THQs for the individuall tested metals for both males and females in all soil types were less than one [Fig. 1 (a), (b)]. This indicates that daily intake of each substance may not lead to a health risk. This further confirmed that organic-plant-based compost is a safe way to improve the growth of *M. oleifera* because it limits the bioavailability of heavy metals in the soil and their accumulation in the leaves (Olaniran et al. 2013). The aluminum THQs decreased with the addition of plant-based compost. Similar results were reported by Ghanim et al. (2023), where the addition of compost to the soil reduced the THQs of heavy metals in pea plants.

### Conclusion and Implications of Using Plant-Based Compost for Clay Soil Amendments in Growing *M. oleifera* Plants

The overall outcome of the study affirms that the addition of plant-based compost to clay soil increases the content and availability of metals in *M. oleifera* leaf biomass. The essential nutrient elements Ca, K, Mg, P and S found at high concentrations in the *M. oleifera* biomass studied are major nutrients needed for humans and animals. The concentrations of these nutrients significantly increased with the addition of compost to the soil. However, the addition of plant-based compost did not significantly increase the availability or accumulation of trace metals such as Cr and Co, which are needed at relatively low concentrations for plant growth. Thus, plant-based compost is recommended for increasing essential elements in the soil and *M. oleifera* leaves and for suppressing trace and/or heavy metals that could be present in the soil. In this study, it was discovered that addition of 30% plant compost to clay soil gave the highest amount of major nutrients of Ca, K, Mg, P and S. This composition is therefore recommended for improved nutrients in *M. oleifera* leaves at the *M. oleifera* farms grown in clay soils. In addition, the nutrient concentration of *M. oleifera* leaves grown under plant-based compost soil amendments is recommended for use as a nutrient supplement for both humans and animals. Metals such as Al, Co, Cr, Fe, Mn and Zn are known to have toxic effects on the human body; therefore, caution must be taken when consuming leaf powder. The noncarcinogenic test based on the THQ values confirmed that *M. oleifera* in all the soil amendments in the present study was safe for consumption. However, comprehensive studies are needed before plant-based composts are used to increase the nutrient content of *M. oleifera* biomass, in order to avoid increasing the concentration of trace elements above the maximum permissible limits.

## References

[CR1] Adebayo AG, Akintoye HA, Shokalu AO, Olatunji MT (2017). Soil chemical properties and growth response of Moringa oleifera to different sources and rates of organic and NPK fertilizers. Int J Recycling Org Waste Agric.

[CR2] Adugna G (2016). A review on impact of compost on soil properties, water use and crop productivity. Acad Res J Agricultural Sci Res.

[CR3] Akinola R, Pereira LM, Mabhaudhi T, De Bruin FM, Rusch L (2020) A review of indigenous food crops in Africa and the implications for more sustainable and healthy food systems. Sustainability. 24;12(8):3493. 10.3390/su12083493 10.3390/su12083493 PMC7116648 33520291

[CR4] Alan M, Kara D (2019). Comparison of a new sequential extraction method and the BCR sequential extraction method for mobility assessment of elements around boron mines in Turkey. Talanta.

[CR5] Ammari TG, Al-Omari Q, Abbassi BE (2012) Composting sewage sludge amended with different sawdust proportions and textures and organic waste of food industry–assessment of quality. Environmental Technology. 1;33(14):1641-9. 10.1080/09593330.2011.641589 10.1080/09593330.2011.641589 22988624

[CR6] Areo OM, Njobeh PB (2021). Risk assessment of heavy metals in rooibos (Aspalathus linearis) tea consumed in South Africa. Environ Sci Pollut Res.

[CR7] Asiedu-Gyekye IJ, Frimpong-Manso SA, Awortwe C, Antwi DA, Nyarko AK (2014). Micro-and macro elemental composition and safety evaluation of the nutraceutical Moringa oleifera leaves. J Toxicol.

[CR8] Aslam M (2005). Mineral composition of *Moringa oleifera* leaves and pods from different regions of Punjab, Pakistan. Asian J Plant Sci.

[CR9] Bhattacharya A, Tiwari P, Sahu PK, Kumar S (2018) A review of the phytochemical and pharmacological characteristics of *Moringa oleifera*. Journal of Pharmacy and Bioallied Sciences. 1;10(4):181 – 91. 10.4103/jpbs.jpbs_126_18 10.4103/JPBS.JPBS_126_18 PMC6266645 30568375

[CR10] Bożym M (2017) The study of heavy metals leaching from waste foundry sands using a one-step extraction. In E3S Web of Conferences 19: 02018). 10.1051/e3sconf/20171902018

[CR11] Brilhante RS, Sales JA, Pereira VS, Castelo DD, de Aguiar Cordeiro R, de Souza Sampaio CM, Paiva MD, Dos Santos JB, Sidrim JJ, Rocha MF (2017) Research advances on the multiple uses of *Moringa oleifera*: A sustainable alternative for socially neglected population. Asian Pacific Journal of Tropical Medicine. 1;10(7):621 – 30. 10.1016/j.apjtm.2017.07.002 10.1016/j.apjtm.2017.07.002 28870337

[CR12] Celik I, Ortas I, Kilic S (2004) Effects of compost, mycorrhiza, manure and fertilizer on some physical properties of a Chromoxerert soil. Soil and Tillage Research. 1;78(1):59–67. 10.1016/j.still.2004.02.012

[CR13] Chibuike GU, Obiora SC (2014). Heavy metal polluted soils: effect on plants and bioremediation methods. Appl Environ Soil Sci.

[CR14] Crinnion WJ (2010) Organic foods contain higher levels of certain nutrients, lower levels of pesticides, and may provide health benefits for the consumer. Alternative Medicine Review. 1;15(1) 20359265

[CR15] Duong TT, Penfold C, Marschner P (2012). Amending soils of different texture with six compost types: impact on soil nutrient availability, plant growth and nutrient uptake. Plant Soil.

[CR16] Ghanim AA, Shah MA, Alam M, Khan A, Khan MA, Rahman S, Alsaiari MA, Jalalah M, Khan MK, Irfan M, Hussain Z (2023). The influence of compost amendments on bioaccumulation of potentially toxic elements by pea plant cultivated in mine degraded soils. Arab J Geosci.

[CR17] Gopalakrishnan L, Doriya K, Kumar DS (2016) *Moringa oleifera*: A review on nutritive importance and its medicinal application. Food Science and Human Wellness. 1;5(2):49–56. 10.1016/j.fshw.2016.04.001

[CR18] Grosshagauer S, Pirkwieser P, Kraemer K, Somoza V (2021). The future of Moringa foods: a food chemistry perspective. Front Nutr.

[CR19] Haouvang LC, Albert N, Martin Y, Mbaiguinam M (2017) Growth response of *Moringa oleifera* Lam. as affected by various amounts of compost under greenhouse conditions. Annals of Agricultural Sciences. 1;62(2):221-6. 10.1016/j.aoas.2017.12.004

[CR20] Herencia JF, Ruiz JC, Morillo E, Melero S, Villaverde J, Maqueda C (2008) The effect of organic and mineral fertilization on micronutrient availability in soil. Soil Science. 1;173(1):69–80. 10.1097/ss.0b013e31815a6676

[CR21] Ho TT, Le TH, Tran CS, Nguyen PT, Thai VN, Bui XT (2022). Compost to improve sustainable soil cultivation and crop productivity. Case Stud Chem Environ Eng.

[CR22] Hocking PJ (1994) Dry-matter production, mineral nutrient concentrations, and nutrient distribution and redistribution in irrigated spring wheat. Journal of Plant Nutrition. 1;17(8):1289 – 308. 10.1080/01904169409364807

[CR23] Hu C, Xia X, Chen Y, Han X (2018). Soil carbon and nitrogen sequestration and crop growth as influenced by long-term application of effective microorganism compost. Chil J Agricultural Res.

[CR24] Huang M, Zhu Y, Li Z, Huang B, Luo N, Liu C, Zeng G (2016). Compost as a soil amendment to remediate heavy metal-contaminated agricultural soil: mechanisms, efficacy, problems, and strategies. Water Air Soil Pollut.

[CR25] Industrial Development Corporation of South Africa (IDC) (2019) Growing and agro-processing of *Moringa oleifera* with commercial potential in South Africa. LUHLAZA integrated sustainable solutions. Retrieved 2019 Jul 7, from https://www.idc.co.za/wp-content/uploads/2019/06/Luhlaza-Iss_Moringa-Research-Study_Final-Report.pdf

[CR26] Jakubus M (2016) Estimation of phosphorus bioavailability from composted organic wastes. Chemical Speciation & Bioavailability. 1;28(1–4):189 – 98. 10.1080/09542299.2016.1227687

[CR27] Khwairakpam M, Bhargava R (2009). Vermitechnology for sewage sludge recycling. J Hazard Mater.

[CR28] Konmy BB, Olounladé PA, Azando EB, Hounzangbé-Adoté SE (2016). A review on phytochemistry and pharmacology of *Moringa oleifera* leaves (Moringaceae). J Pharmacognosy Phytochemistry.

[CR29] Kumar RR, Park BJ, Cho JY (2013). Application and environmental risks of livestock manure. J Korean Soc Appl Biol Chem.

[CR30] Leone A, Spada A, Battezzati A, Schiraldi A, Aristil J, Bertoli S (2015). Cultivation, genetic, ethnopharmacology, phytochemistry and pharmacology of *Moringa oleifera* leaves: an overview. Int J Mol Sci.

[CR31] Liu YY, Ukita M, Imai T, Higuchi T (2006) Recycling mineral nutrients to farmland via compost application. Water Science and Technology. 1;53(2):111-8. 10.2166/wst.2006.044 10.2166/wst.2006.044 16594329

[CR33] Mahmood KT, Mugal T, Haq IU (2010) Moringa oleifera: a natural gift-A review. Journal of Pharmaceutical Sciences and Research. 1;2(11):775

[CR32] Manungufala TE, Chimuka L, Maswanganyi BX (2008) Evaluating the quality of communities made compost manure in South Africa: A case study of content and sources of metals in compost manure from Thulamela Municipality, Limpopo province. Bioresource Technology. 1;99(5):1491-6. 10.1016/j.biortech.2007.02.006 10.1016/j.biortech.2007.02.006 17418564

[CR34] Mashamaite CV, Pieterse PJ, Mothapo PN, Phiri EE (2021). Moringa oleifera in South Africa: a review on its production, growing conditions and consumption as a food source. South Afr J Sci.

[CR35] Meemken EM, Qaim M (2018). Organic agriculture, food security, and the environment. Annual Rev Resource Econ.

[CR36] Ngakou A, Haouvang LC, Mbaiguinam M, Uke P, Issa M (2020) Changes in pigment contents and nutritional components of Moringa oleifera Lam. as impacted by different feedstuff compost receipts. Compost Science & Utilization. 1;28(3–4):147 – 57

[CR37] Nuapia Y, Chimuka L, Cukrowska E (2018) Assessment of heavy metals in raw food samples from open markets in two African cities. Chemosphere 1. 10.1016/j.chemosphere.2017.12.134. 196:339 – 46 10.1016/j.chemosphere.2017.12.134 29310070

[CR38] Okem A, Southway C, Stirk WA, Street RA, Finnie JF, Van Staden J (2014). Heavy metal contamination in South African medicinal plants: a cause for concern. South Afr J Bot.

[CR39] Olaniran AO, Balgobind A, Pillay B (2013). Bioavailability of heavy metals in soil: impact on microbial biodegradation of organic compounds and possible improvement strategies. Int J Mol Sci.

[CR40] Omara AE, Hafez EM, Osman HS, Rashwan E, El-Said MA, Alharbi K, Abd El-Moneim D, Gowayed SM (2022). Collaborative impact of compost and beneficial rhizobacteria on soil properties, physiological attributes, and productivity of wheat subjected to deficit irrigation in salt affected soil. Plants.

[CR41] Pakade V, Cukrowska E, Chimuka L (2013). Metal and flavonol contents of Moringa oleifera grown in South Africa. South Afr J Sci.

[CR42] Ptistišek N, Milačič R, Veber M (2001). Use of the BCR three-step sequential extraction procedure for the study of the partitioning of cd, pb and zn in various soil samples. J Soils Sediments.

[CR43] Sahito OM, Afridi HI, Kazi TG, Baig JA (2015) Evaluation of heavy metal bioavailability in soil amended with poultry manure using single and BCR sequential extractions. International Journal of Environmental Analytical Chemistry. 2;95(11):1066-79. 10.1080/03067319.2015.1078800

[CR44] Salimi M, Amin MM, Ebrahimi A, Ghazifard A, Najafi P (2012). Influence of electrical conductivity on the phytoremediation of contaminated soils to cd 2 + and zn 2+. Int J Environ Health Eng.

[CR46] Sarwar M, Ali A, Nouman W, Arshad MI, Patra JK (2017). Compost and synthetic fertilizer affect vegetative growth and antioxidants activities of Moringa oleifera. Int J Agric Biology.

[CR45] Sarwar M, Patra JK, Ali A, Maqbool M, Arshad MI (2020). Effect of compost and NPK fertilizer on improving biochemical and antioxidant properties of Moringa oleifera. South Afr J Bot.

[CR47] Siddhuraju P, Becker K (2003) Antioxidant properties of various solvent extracts of total phenolic constituents from three different agroclimatic origins of drumstick tree (*Moringa oleifera* Lam.) leaves. Journal of Agricultural and Food Chemistry. 9;51(8):2144-55. 10.1021/jf020444+ 10.1021/jf020444+ 12670148

[CR48] Sung M, Lee CY, Lee SZ (2011) Combined mild soil washing and compost-assisted phytoremediation in treatment of silt loams contaminated with copper, nickel, and chromium. Journal of Hazardous Materials. 15;190(1–3):744 – 54. 10.1016/j.jhazmat.2011.03.113 10.1016/j.jhazmat.2011.03.113 21531509

[CR49] Sungur A, Soylak M, Ozcan H (2014). Investigation of heavy metal mobility and availability by the BCR sequential extraction procedure: relationship between soil properties and heavy metals availability. Chem Speciat Bioavailab.

[CR50] Tshabalala T, Ncube B, Moyo HP, Abdel-Rahman EM, Mutanga O, Ndhlala AR (2020). Predicting the spatial suitability distribution of Moringa oleifera cultivation using analytical hierarchical process modelling. South Afr J Bot.

[CR51] Van Herck P, Van der Bruggen B, Vogels G, Vandecasteele C (2000) Application of computer modelling to predict the leaching behaviour of heavy metals from MSWI fly ash and comparison with a sequential extraction method. Waste Management. 1;20(2–3):203 – 10. 10.1016/s0956-053x(99)00321-9

[CR52] Verma SL, Penfold C, Marschner P (2013). Mobilisation of rock phosphate by surface application of compost. Biol Fertil Soils.

[CR53] Vouillamoz J, Milke MW (2001) Effect of compost in phytoremediation of diesel-contaminated soils. Water Science and Technology. 1;43(2):291-5. 10.2166/wst.2001.0102 11380193

[CR54] Yogev A, Raviv M, Hadar Y, Cohen R, Katan J (2006). Plant waste-based composts suppressive to diseases caused by pathogenic Fusarium oxysporum. Eur J Plant Pathol.

[CR55] You Y, Rankin SC, Aceto HW, Benson CE, Toth JD, Dou Z (2006). Survival of Salmonella enterica Serovar Newport in manure and manure-amended soils. Appl Environ Microbiol.

[CR56] Zhao XQ, Shen RF (2018). Aluminum–nitrogen interactions in the soil–plant system. Front Plant Sci.

[CR57] Zheljazkov VD, Warman PR (2004) Phytoavailability and fractionation of copper, manganese, and zinc in soil following application of two composts to four crops. Environmental Pollution. 1;131(2):187 – 95. 10.1016/j.envpol.2004.02.007 10.1016/j.envpol.2004.02.007 15234085

